# Kelp-stone ratio and bed roughness control green gravel stability under hydrodynamic forces

**DOI:** 10.1371/journal.pone.0345763

**Published:** 2026-04-20

**Authors:** Maike Paul, Nils B. Kerpen, Armin Moghimi

**Affiliations:** Leibniz University Hannover, Ludwig-Franzius-Institute of Hydraulic, Estuarine and Coastal Engineering, Germany; National Cheng Kung University, TAIWAN

## Abstract

Kelp forests play a crucial role in marine ecosystems, providing habitat, food, and shelter for a variety of marine organisms. However, these ecosystems are facing significant threats, prompting the need for effective restoration strategies. One promising method is the use of “Green Gravel”, where kelp spores are cultivated on small stones for subsequent deployment in the ocean. The success of this method strongly depends on the kelp attaching firmly to the ground before the kelp-stone systems get displaced to unsuitable locations by hydrodynamic forcing. Here we systematically quantified the hydrodynamic and bed conditions leading to initiation of motion of kelp-stone systems in the laboratory. For full control on kelp dimensions, surrogates were used. Critical shear stress negatively correlated with kelp size, and bed roughness had a stronger influence on the initiation of motion than stone dimensions or bed slope. We introduce the Shields parameter with a stability correction, highlighting its strong correlation with kelp frontal area and stone diameter, and providing a powerful unifying metric for predicting the onset of movement under diverse conditions. Additionally, subtle movement of the kelp-stone systems prior to displacement was observed, potentially preventing attachment even at hydrodynamic conditions below the thresholds for initiation of motion. Understanding the mechanistic processes leading to motion of kelp-stone systems helps to improve restoration methods and thus contributes to the broader objectives of marine habitat conservation.

## Introduction

Kelp forests are crucial to coastal biodiversity and they provide additional services such as wave attenuation and retention of suspended sediment [1–3]. Furthermore, kelp forests have been found to act as carbon sinks, which would contribute to climate change mitigation [4,5]. Despite their ecological importance, a global decline in kelp forests has been observed, which is primarily attributed to climate change [6,7]. The pressing need to restore these vital ecosystems has led to the exploration of innovative methods such as the Green Gravel method. This approach involves growing kelp spores on small stones in laboratories, which are then deployed in the ocean to encourage growth and habitat restoration [8]. The method presents several advantages over traditional restoration methods, which are labour-intensive, costly, and dependent on divers to plant kelp on the seabed [9]. In contrast, Green Gravel allows for large-scale planting which requires less resources and potentially facilitates the growth of resilient kelp varieties [9].

Despite the promising potential of the Green Gravel method, several challenges remain. Recent studies [see [9] for an overview] have highlighted existing uncertainties associated with water movement, particularly in wave-exposed environments. However, the method's success is contingent upon the ability of the stones to remain stable on the seabed, allowing the kelp to anchor effectively [9]. Research indicates that the stability of the colonised stones is substantially influenced by their size and weight [10]. Alsuwaiyan et al. [11] have demonstrated that kelp can adhere to various stone surfaces, although they did not observe how different stone textures and shapes affect underwater stability. A more recent study found that smaller stones, ranging from 3–5 cm and weighing 50–100 g, tend to be displaced more quickly than larger ones, which measured between 6.4–11 cm and weighed 200–300 g [10]. Moreover, Chemello et al. [12] observed that light intensity positively influences kelp growth rates, thus benefitting kelp development in low water depths. Given that wave forces at the bed increase with decreasing water depth, a trade-off may exist between kelp growth and exposure to increased wave forces and thus negative effects on stability in low water depths. Consequently, comprehensive evaluations considering stone and substrate characteristics, alongside local hydrodynamic conditions, are essential for refining the Green Gravel approach. Together with understanding of the specific number of stones needed for successful restoration such evaluation results could enhance method efficacy [8].

Calculating the onset of movement for sediment or small stones is inherently complex due to the variability in environmental conditions and material properties. Although numerous studies have attempted to model these dynamics, no single formula is universally applicable across all conditions. This variability in predictions introduces significant uncertainties. Previous research has documented the onset of movement for both sediment and stones of varying sizes with attached mature algae [13,14], yet the impact of algae particularly in its juvenile stage has not been fully quantified. Prior to the development of the Green Gravel method, studies had sporadically examined the movement onset of stones with attached kelp. Standard computational approaches, such as those proposed by Hjulström [15] and Shields [16], have been predominantly employed, though these models were not initially designed to account for wave action or the presence of attached algae. As such, there is a need for refined calculation methods that can account for these additional variables to improve the predictive accuracy necessary for applications like the Green Gravel method.

This study focuses on investigating the impact of hydrodynamic forces on the movement behaviour of stones with attached kelp surrogates. To cover a broad range of natural conditions and yet disentangle individual processes, two experiments under controlled flow and wave conditions, respectively, were conducted in the laboratory. By using geotextile to simulate kelp we seek to assess how stone size, shape, and the size of the attached kelp influence the initiation of stone motion. We aim to develop a reliable predictive method for calculating the movement onset of stones with kelp under a range of hydrodynamic conditions and thus contribute to determining site suitability for kelp restoration. Although the experimental setup was designed based on conditions around Heligoland – which represents the only natural kelp population in German waters and is currently a focal point for afforestation efforts [17] – this work will provide general insights to optimize the Green Gravel method for successful large-scale applications, ultimately enhancing restoration and afforestation efforts through a deeper understanding of the dynamics at play.

## Methods

### Kelp surrogates

To have full control over the kelp dimensions, surrogates were used, which were attached to individual stones. Following sieving of a random diabase stone sample, two size classes of stones were used (11.2–16 mm, referred to as $D_{n}=\text{1}\text{6}\text{mm}$ and 16–22 mm, referred to as $D_{n}=\text{2}\text{2}\text{mm}$). For the flow experiment (see Experimental Setup – flow conditions) 13 stones were selected randomly from the smaller stone class, ensuring that the selection process was unbiased and did not favour any particular size, shape, or other characteristic. Nevertheless, measurement of the stone dimensions yielded a $D_{n}=\text{1}\text{3}\text{mm}$ for the stone sample used in the flow experiment and was thus used subsequently. While for the wave experiment (see Experimental Setup – wave conditions) 150 stones from each class were used; no control of $D_{n}$ was conducted for these samples based on their large size. For each stone all three dimensions were measured with a digital calliper gauge (Kraftixx) and their weight was determined with a balance (Sartorius Practum) to the nearest 0.01 g. Their volume was determined by water displacement [18] and mass density was determined as 2,988.8 kg/m^3^. Axial dimensions were used to classify the stone’s shape [19], grouping them into four categories (oblate, equiaxial, triaxial, prolate) depending on the ratio between their axial dimensions which gives an indication for their sphericity [20].

The artificial kelp was produced from geotextile, which was found to be a good match for *Laminaria sp.* [21]. Although the (bio)mechanical traits differ between the used geotextiles and adult *Laminaria sp.* (Table 1), a sufficient similarity is assumed based on the large natural variability of biomechanical traits of *Laminaria sp.* [25] and visual comparison of specimen posture and motion under unidirectional flow (M. Paul, pers. obs.).

**Table 1:** (Bio)mechanical traits of used surrogate material and selected *Laminaria* *sp.* for comparison. Mean values are given ± standard deviation. n is sample size.

Material	Thickness	Tissue density*	Flexural rigidity	Location	Source
	x10^-3^ m	kg/m^3^	x10^-4^ Nm^2^		
*Laminaria digitata*, blade base	0.8 ± 0.13	1001.5 ± 102.7	54.2 ± 27.6^a^	Sletvik, Norway	Paul et al. [22]
*Laminaria digitata*, blade tip	0.5 ± 0.05	1001.5 ± 102.7	51.9 ± 30.4^a^	Sletvik, Norway	Paul et al. [22]
*Laminaria hyperborea*, blade base	1.15 ± 0.4	1126.9 ± 330.3	0.156 ± 0.104^b^	MarGate near Heligoland, Germany	Sarma et al. [23]
Geotextile, flow experiments	0.8	1093.8	110.6 ± 45.3^a^	N/A	Paul and Henry [21]
Geotextile, wave experiments	1.13 ± 0.1	747.4	0.071 ± 0.029^b^	N/A	Paul et al. [24] n=5

*In case of geotextile, the wet tissue density is used to represent submerged conditions; ^a^ values obtained with a 3-point bending test, ^b^ values obtained with a cantilever test following the method of Henry [25].

Surrogates were cut in rectangles of 2 x 10 cm and one short side was glued to a stone with superglue. As a consequence, the surrogate’s base (up to 5 mm) stiffened due to the uptake of glue. In the course of the experiments, surrogates were then cut from the top to achieve smaller surrogate sizes.

### Experimental Setup – flow conditions

**Fig 1:**
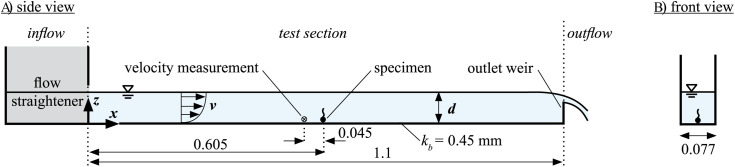
Experimental setup for flow experiment in (A) side view and (B) front view (dimensions in meter).

The experiments to determine the critical shear stresses under unidirectional flow were conducted in a flume with a working section of 1.13 m length, 7.7 cm width and 15 cm height (Fig 1). The bottom consists of an iron plate (bed roughness $k_{b}=\text{0}.\text{2}\text{5}$ mm). Flow was generated with a pump capable of generating a maximum flow rate of 1.6 litres per second. This unidirectional flow was dammed downstream by an adjustable overflow weir, which was used to control the flow velocity and achieve the required water depth. Water level ranged from 4.8≤d≤11.05 cm. A sponge was installed at the flow inlet to prevent turbulence upon water entry and ensure a steady flow through the flume. Specimens were tested individually and placed in the centre of the flume at a distance 60.5 cm downstream of the flow inlet, with each stone set in a predefined orientation consistent throughout the trials.

Flow velocities were measured using an Acoustic Doppler Velocimeter (ADV, Vectrino Profiler from Nortek). It was set to record velocity profiles 10 mm above the bed with a vertical resolution of 1 mm and a sampling rate of 100 Hz. The ADV was positioned 45 mm upstream of the specimens allowing for the assessment of flow velocities acting on them.

At the start of each series of measurements, the critical flow conditions for the specimen with the maximum surrogate area (20 cm²) were recorded. The surrogate was oriented to ensure that the entire area was subjected to flow. The critical condition is defined by the stone maintaining its position during measurement until it moves as velocity increases. Movements that do not lead to a positional change (e.g., rocking or tilting) were not considered as surpassing the critical condition.

Once the specimen’s position was deemed stable, the weir was gradually lowered. According to the continuity equation, this reduced weir height increased flow velocity. The weir was lowered in approximately 4 mm increments. If the stone moved after lowering the weir, the prior stable velocity was identified as the critical condition. Each measurement lasted three minutes, commencing either at the start of the trial or with further weir adjustments. If movements occurred before three minutes elapsed, the measurement concluded at the end of the three-minute interval, ensuring the applicability of the average value and identifying the critical moment in data analysis. Post measurement, the surrogate height was reduced consecutively by 2 cm each time until only the stone was evaluated. This procedure was repeated for all 13 stones, and their respective critical conditions were determined.

### Experimental setup – wave conditions

**Fig 2:**
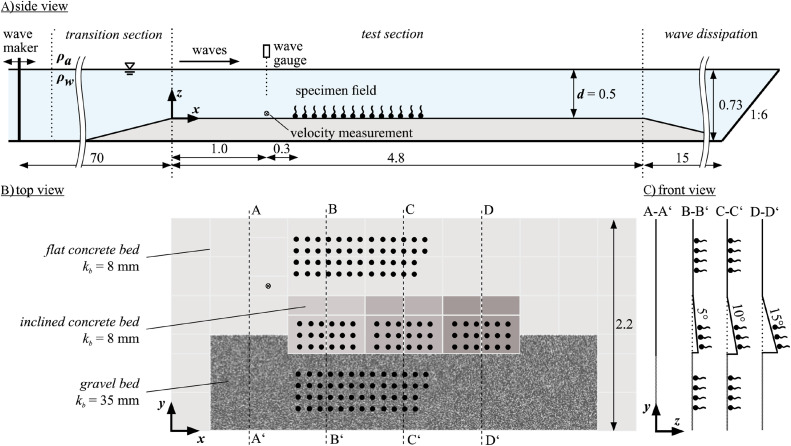
Experimental setup for wave experiment in (A) side view, (B) top view depicting the different bed types next to each other and (C) front view depicted as cross sections along the dashed lines indicated in B. k_b_ is bed roughness. (dimensions in meter).

The experiments were conducted in the wave flume (110 m long, 2.2 m wide) at Ludwig-Franzius-Institute of Leibniz University Hannover. The test area within the flume was positioned at the observation window starting 70 m from the wave maker. Euro pallets were laid across the test section as the foundation for the experimental setup, allowing for substructure water flow. On top of the pallets, two layers paving slabs (40 cm x 40 cm x 5 cm) were installed. Overall, the setup consisted of three sections (Fig 2B): (1) Flat concrete bed: A flat concrete area ($k_{b}=\text{8}$ mm) was created with additional paving slabs laid with their rough sides up, forming an 80 cm wide and 180 cm long area. (2) Inclined concrete bed: Another section was constructed with tilted slabs ($k_{b}=\text{8}$ mm), using wooden chocks to create slopes of 5°, 10°, and 15°. This range of slope angles was chosen to cover the majority of angles observed at MarGate near Heligoland [26]. Each sloped section was 60 cm wide and 80 cm long and with a slope increase in the direction of wave travel. (3) Gravel bed: An 80 cm wide gravel layer ($d_{\text{9}\text{0}}=k_{b}=\text{3}\text{5}$ mm) was placed alongside the inclined slabs in a way that its mean height was level with the mean concrete bed. The gravel, mixed with epoxy resin to prevent movement, was evenly distributed over geotextile support. This design was chosen to mimic rough rocky surfaces similar to those observed at MarGate near Heligoland [26].

For wave management, limestone blocks formed a transition slope to guide wave flow effectively.

The preparation included marking stone positions (circles with 5 cm diameter) with their centres spaced 13.3 cm apart with white spray paint on the different substrate areas. Black waterproof markers enhanced visibility on concrete substrates.

A Microsonic MIC130−4 ultrasonic sensor (accuracy: 0.18 mm) was mounted from the channel wall 30 cm in front of the first specimen positions (Fig 2A). It recorded water surface elevations to determine wave heights and periods at a sampling rate of 300 Hz. Data acquisition was synchronised with video recordings from two Logitech webcams, capturing lateral views focusing on a single stone and providing an overhead perspective from 6 m above the channel floor. The camera setups enabled comprehensive monitoring of stone and kelp movement under varying wave conditions.

The wave boundary conditions were derived from the Heligoland North tide gauge data [27] for the period from January 1^st^ to December 31^st^ 2021. They represent an average significant wave height of $H_{s}=\text{1}.\text{0}\text{1}\text{m}$ and a relatively frequently recorded wave period of $T_{p}\approx \text{5}\text{s}$. Using linear wave theory, the resulting near-bed velocity at a typical kelp settlement depth of d=10m was calculated as $u_{b}=\text{0}.\text{2}\text{3}\text{4}\text{m}/\text{s}$ according to equation (5). The wave flume boundary conditions were configured to achieve comparable near-bed flow velocities, with a constant wave period of T=5s. This was achieved with a water depth of d=0.5m and a wave height of H=0.11m. Hence, regular waves were generated with a constant wave period of $T_{m}=\text{5}\text{s}$ and varying wave heights ($\text{0}.\text{0}\text{1}\text{m}\leq H_{m}\leq \text{0}.\text{2}\text{2}\text{m}$).

Each wave train consisted of 24 waves to minimise interaction with waves reflecting from the flume’s end. Experiments were divided into two groups based on the stone classes. For each stone class testing began with maximum surrogate area ($A_{k}=\text{2}\text{0}$ cm²). Tests began with a 1 cm wave height, increasing in 1 cm intervals until most specimens moved. Then, surrogate height was clipped to achieve surrogate heights of 10, 8, 4, 2 and 1 cm. Tests repeated with each surrogate height until only evaluating the stone. Maximum wave height was 22 cm for the larger stone class without surrogate.

### Specimen stability and resistance analysis

The load on and strength of the specimen were analysed to derive its stability on the bed. The load on the specimen is expressed by the critical shear stress $\tau^{*}$ which is required to initiate a motion of the specimen. This critical shear stress is a function of the critical shear velocity $u^{*}$ (velocity over the bottom, when at initiation of motion) and the density of the fluid $\rho_{f}$.


$$\tau^{*}=u^{*\text{2}}\rho_{f}$$ (1)

The resistance of the specimen is expressed by the nominal stone diameter $D_{n}$, the acceleration of gravity g and the density of the surrogate $\rho_{s}$ reduced by the density of the fluid $\rho_{f}$. The critical Shields parameter $\theta^{*}$ takes the relation of the critical load and the strength of the specimen into account, where it is proportional to the ratio of fluid force on the particle to the weight of the particle


$$\theta^{*}=\frac{load}{resistence}=\frac{u^{*\text{2}}\rho_{f}}{\left(\rho_{s}-\rho_{f}\right)gD_{n}}=\frac{\tau^{*}}{\left(\rho_{s}-\rho_{f}\right)gD_{n}}(-)$$ (2)

**Fig 3:**
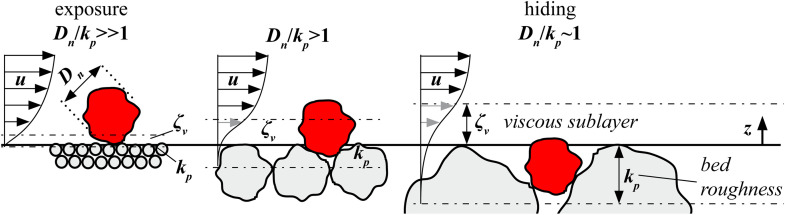
The influence of the ratio of stone size ${𝐃}_{n}$ and absolute bed roughness ${𝐤}_{b}$ on the transportability of stones. Influence of the bed roughness ${𝐤}_{b}$ on the viscous sublayer $\zeta_{v}$ and the near-bed flow profile 𝐮 under steady flow condition.

To account for potential hiding and exposure effects the relation of the absolute bed roughness $k_{b}$ to the nominal diameter $D_{n}$ of the specimen’s stone is taken into account. For $D_{n}/k_{b}\gg \text{1}$ the stone is fully exposed to the near-bed flow velocity u (Fig 3). The majority of the stone is exposed to the flow outside the viscous sublayer of the bed. For $D_{n}/k_{b}>\text{1}$ the thickness of the viscous sublayer $\zeta_{v}$increases and leads to reduced flow intensity on the stone. Additionally, the stability of the stone against rolling increases. For $D_{n}/k_{b}\leq \text{1}$ two aspects become important. First, with decreasing value $D_{n}/k_{b}$ the stone is fully located in the viscous sublayer with significant lower flow velocity. Additionally, the stone is hiding within the bed roughness and almost not exposed to the bottom flow velocity. This leads to a further increased stability against rolling compared to larger $D_{n}/k_{b}$ values. The value $D_{n}/k_{p}$ can be interpreted as a stability factor influencing the resistance term of the Shields parameter.

### Data analysis

For the ADV data of the flow experiments, only the downstream velocity component was considered. Poor quality data was removed from the time series by excluding data points with a Signal-to-Noise-Ratio <15 dB and a correlation <90%. The remaining data was used to compute a mean value for each position in the velocity profile. Visual inspection showed that vertical velocity distributions followed a logarithmic profile in all cases. Subsequently, data from 1 cm above the bed was used to compute the critical shear velocity $\overset{*}{\underset{flow}{u}}$ according to:


$$\frac{v(y)}{\overset{*}{\underset{flow}{u}}}=\frac{\text{1}}{\kappa }ln\frac{\overset{*}{\underset{flow}{u}}y}{\nu }+\text{5}.\text{5}$$ (3)

With v(y) being the mean velocity at height y, κ=0.4 being the von Kármán constant and ν being the kinematic viscosity ($\text{1}.\text{0}\text{0}\text{2}\cdot {\text{1}\text{0}}^{-\text{6}}$ m²/s). This was then used to compute the critical shear stress with eq. 2.

For the wave experiment, the video footage was analysed to determine the onset of specimen displacement starting systematically with the smallest wave height and progressing through the conditions. A specimen was marked as moved if it was fully or partially outside the predefined starting circles at the end of a wave sequence. Once it was marked as moved, it kept this label for all following wave conditions. It should be noted that all 150 specimens were tested simultaneously under each wave condition. This approach follows the concept of sample replication from field ecology [28] and is deemed feasible and appropriate in this experimental setup as the work was carried out with inert material. In our experiments, only the physical conditions were relevant, and we maintained full control over these parameters, while potential effects of other environmental conditions were not applicable.

Time series of water surface displacement corresponding to the wave conditions at which individual specimens were marked as moved for the first time were used for analysis. Mean wave height $H_{m}$ was calculated for the 24 waves in the wave train using the distance between the mean height of wave crests and troughs, respectively. Linear wave theory was then applied to calculate the resulting critical shear velocity $\overset{*}{\underset{wave}{u}}$:


$$\overset{*}{\underset{wave}{u}}=\frac{H_{m}\omega }{\text{2}sinh\left(kd\right)}$$ (4)

Where $\omega =\frac{\text{2}\pi }{T_{m}}$ is the angular frequency, $k=\frac{\text{2}\pi }{L_{m}}$ is the wave number, d is water depth (0.5 m) and $L_{m}$ is mean wave length, which was derived from mean wave period $T_{m}$ via the dispersion relation. The critical wave shear stress was then derived by applying the Swart-formula [29], which defines the wave friction factor as a function of the relative bed roughness:


$$\overset{*}{\underset{wave}{\tau }}=\text{0}.\text{5}\rho_{f}f_{w}\overset{*\text{2}}{\underset{wave}{u}}$$ (5)

with


$$f_{w}=exp\left[-\text{5}.\text{977}+\text{5}.\text{213}{\left(\frac{\overset{*}{\underset{wave}{u}}T_{m}}{\text{2}\pi k_{b}}\right)}^{-\text{0}.\text{194}}\right]$$ (6)

Where $k_{b}$ is the absolute bed roughness which was set to 8 mm for the concrete bed and to 35 mm for the gravel bed [30].

To investigate how surrogate length influences their dynamic behaviour, deformation was analysed using video recordings of an individual specimen. To extract the shape and position of the surrogate in the videos, the SAM2 (Segment Anything Model, version 2) [31] artificial intelligence (AI) algorithm developed by Meta was employed. Leveraging its prompt-based architecture, SAM2 enabled efficient semi-automatic segmentation while eliminating the need for extensive training and annotated ground truth data. By providing just 3–6 point prompts on the surrogate in only 1–2 key frames, the model dynamically processed entire video sequences, achieving accurate segmentation across all frames. Following segmentation, the deformation of the surrogate was analysed for each considered surrogate length in the X–Z plane over one wave cycle — at normalized times t/T = {0, 2/5, 3/5, 4/5, 1}.

Statistical differences within the dataset were analysed to identify the contribution of the variables surrogate length $l_{k}$, stone dimensions and bed type to the critical shear stress. A one-way Analysis of Variance (ANOVA) was employed to evaluate the statistical differences in the critical shear stress across the slope angles (0°, 5°, 10°, 15°). Additionally, individual one-way ANOVAs were conducted to determine if the critical shear stress varied significantly across stone shapes (oblate, equiaxial, triaxial, prolate) within each bed type (concrete vs gravel) for the surrogate lengths $l_{k}=\text{0}\text{cm}$ and 10 cm. This helped identify the influence of stone shape independent of bed type. Linear regression models were employed to explore the interaction effects of continuous predictor variables, which were not possible to assess using ANOVA alone. They were applied for quantitatively assessing the contribution (1) of stone weight (used as a proxy for stone size), surrogate length ($l_{k}$) and their interaction on the critical shear stress under wave conditions ($\overset{*}{\underset{wave}{\tau }}$), and (2) of potential interaction effects of slope, stone weight and surrogate length $l_{k}$ on $\overset{*}{\underset{wave}{\tau }}$. Normality was tested with a One-sample Kolmogorov-Smirnov test. Although for selected datasets the test results indicated deviations from normality, the above methods were applied given their robustness to departures from the normality assumption. The significance level for all statistical tests was set at α = 0.05. All data processing and analyses was done in MATLAB R2024b.

## Results

**Fig 4:**
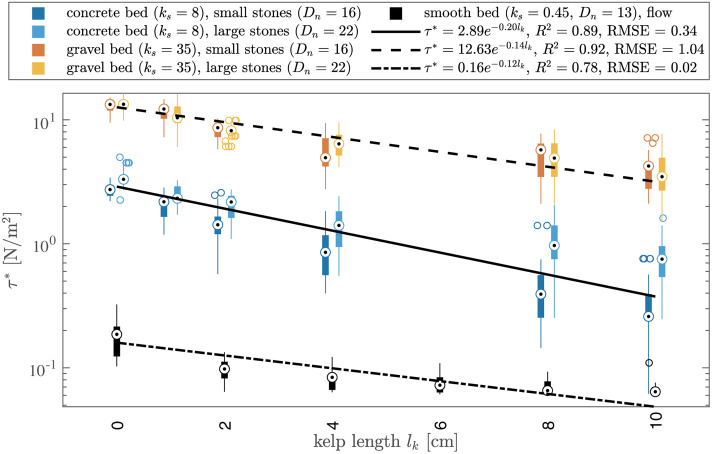
Effect of surrogate length ${𝐥}_{k}$ with constant width ${𝐰}_{k}=\text{2}cm$ on critical shear stress $\tau^{*}$ for concrete, gravel and smooth beds and two different stone sizes. Boxes indicate the interquartile range (IQR), black dots represent the median, whiskers are maximum and minimum values within the 1.5 × IQR of the hinge and outliers are depicted by empty circles.

Overall, the results show that larger surrogates led to lower critical shear stresses $\tau^{*}$, irrespective of nominal stone diameter $D_{n}$ or bed roughness $k_{b}$ (Fig 4), indicating that stones colonised by kelp will move at lower velocities than uncolonized stones. The critical shear stress $\tau^{*}$ required to initiate the movement of a stone with surrogate length $l_{k}=\text{1}\text{0}\text{cm}$ (given a constant surrogate width $w_{k}=\text{2}\text{cm}$ the frontal area is $A_{k}=\text{2}\text{0}\text{c}{\text{m}}^{\text{2}}$) was about 70–85% lower than that of an uncolonized stone. Moreover, the data highlight the effect of bed roughness on critical shear stress (Fig 4). The division by stone classes suggested an effect of stone size in the case of the concrete bed ($k_{b}=\text{8}\text{ mm}$), but not for the gravel bed ($k_{b}=\text{3}\text{5}\text{ mm}$). This was confirmed by linear regression models which were constructed to evaluate the effect of stone weight (as a proxy for stone size) and surrogate length including their interaction on the critical shear stress under waves ($\overset{*}{\underset{wave}{\tau }}$). The model for the concrete bed explained 70% of the variance in $\overset{*}{\underset{wave}{\tau }}$ and indicated a significant influence of surrogate length (p<0.001) and stone weight (p<0.001), while their interaction had no significant effect (p=0.30). The model for the gravel bed had a comparable fit ($R^{\text{2}}=\text{0}.\text{6}\text{5}$) and supported the significant influence of surrogate length on $\overset{*}{\underset{wave}{\tau }}$ (p<0.001), but indicated that neither stone weight (p=0.59) nor their interaction (p=0.39) had an effect on $\overset{*}{\underset{wave}{\tau }}$ in this case.

### Impact of slope

**Fig 5:**
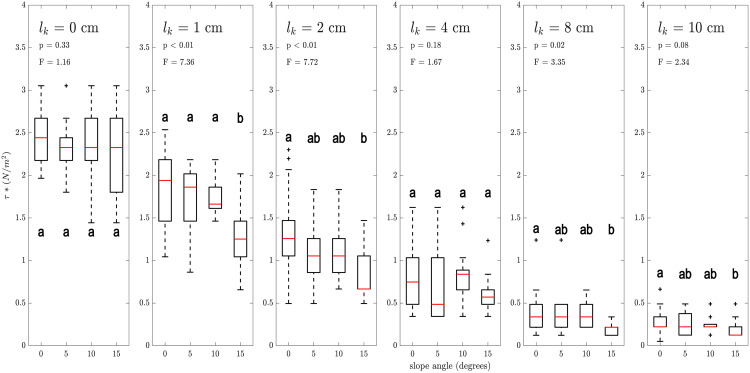
Effect of bed slope angle on critical shear stress on the concrete bed for different surrogate lengths l_k_ as indicated in the panels. Boxes indicate the interquartile range (IQR), red lines in the boxes represent the median, whiskers are maximum and minimum values within the 1.5 × IQR of the hinge and outliers are depicted by crosses. Test statistics of a one-way ANOVA, evaluating the statistical differences in critical shear stress across various slope angles, are given in each panel, letters inside each panel indicate results from a Tukey’s posthoc test. Sample sizes are n = 50 for 0°, n = 17 for 5° and 10°, n = 14 for 15°.

The slope angles (0°, 5°, 10°, 15°) showed little effect on the critical shear stress $\overset{*}{\underset{wave}{\tau }}$ (Fig 5). Specimens on the 15° slope generally moved at lower shear stresses than the specimens on the flat concrete, irrespective of surrogate length, but the effect of intermediate slope angles remained inconclusive. This was confirmed by a linear regression model ($R^{\text{2}}=\text{0}.\text{6}\text{9}$) testing for potential interaction effects of slope, stone weight and surrogate length $l_{k}$ on $\overset{*}{\underset{wave}{\tau }}$. While stone weight and surrogate length had a significant effect on $\overset{*}{\underset{wave}{\tau }}$ (p < 0.001), slope did not (p = 0.29). There was a weak interaction effect between slope and stone weight (p = 0.047), but no other interaction effects existed.

### Impact of stone shape

Overall, the scatter within the data reduced with increasing surrogate size, although this was more prominent under flow conditions than under waves. It suggests that the overall influence of the surrogate on the force balance increased and the effect of stone shape (oblate, equiaxial, triaxial, prolate), which caused the increased scatter for stone only measurements, became negligible in comparison. This was confirmed by the comparison of stone shapes [19] for the wave experiments (see Kelp Surrogates). Individual one-way ANOVAs for surrogate lengths $l_{k}=\text{0}$ and 10 cm and each bed type across the four stone shapes yielded no effect of stone shape (p>0.1). The results did not differ if stone classes ($D_{n}$) were analysed together or separately. Based on this finding, intermediate surrogate lengths ($l_{k}=\text{1};\text{2};\text{4}$ and 8 cm) were not analysed.

### Initiation of motion

**Fig 6:**
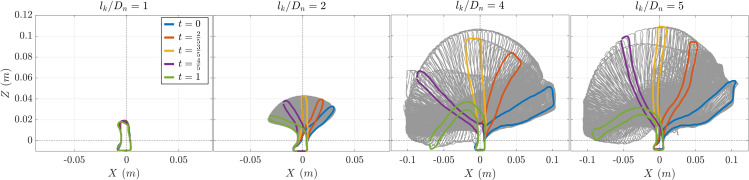
Temporal variation of horizontal displacement X versus vertical position Z during one wave cycle for four surrogate lengths ($\frac{{𝐥}_{k}}{D_{n}}=\text{1}$, 2, 4 and 5). Grey surrogate shapes indicate the positions in each analysed video frame while the coloured shaped highlight surrogate positions at certain steps within a wave period.

The surrogates display a dynamic motion throughout a wave period which increases with increasing surrogate length up to a certain size (Fig 6). For $\frac{l_{k}}{D_{n}}=\text{1}$ stem displacement was minimal, suggesting limited wave impact. At $\frac{l_{k}}{D_{n}}=\text{2}$, noticeable tip deflection indicated the onset of a dynamic response. At higher surrogate lengths, especially $\frac{l_{k}}{D_{n}}=$4 and 5, the oscillation amplitude increased markedly, and a back-and-forth motion synchronized with the wave cycle was observed.

**Fig 7:**
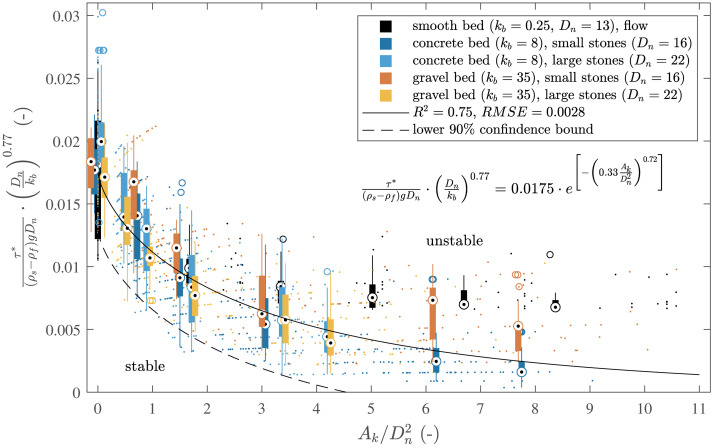
Correlation of the critical Shields parameter including stability correction for hiding and exposure with the relative kelp frontal area. Data for three different levels of bed roughness ${𝐤}_{b}$, 3 different stone diameters ${𝐃}_{n}$, 6 different kelp frontal areas ${𝐀}_{k}$ and 50 specimens/test (13 for the smooth bed). Small dots indicate data for each individual specimen. Boxes indicate the interquartile range (IQR), black dots represent the median, whiskers are maximum and minimum values within the 1.5 × IQR of the hinge and outliers are depicted by empty circles. The median fit (eq. (7), solid line) and the corresponding 90% lower confidence bound (dashed line) are given.


To assess the onset of spatial stone displacement, the critical shear stress $\tau^{*}$ was converted to the dimensionless Shields parameter, combined with a stability factor accounting for nominal stone diameter $D_{n}$ and bed roughness $k_{b}$. This aligned all data along a single curve when plotted against the ratio of surrogate frontal area $A_{k}$ to stone diameter $D_{n}$ (Fig 7). A non-linear least squares fitting method was applied to model the data with an exponential decay function of the form:

$$\underset{Sc\text{h}ieldsparameter}{\underbrace{\frac{\tau^{*}}{\left(\rho_{s}-\rho_{f}\right)gD_{n}}}}\cdot \underset{\begin{array}{c} stability\\ factor \end{array}}{\underbrace{{\left(\frac{D_{n}}{k_{b}}\right)}^{\text{0}.\text{77}}}}=\text{0}.\text{0175}\cdot e^{\left[{-\left(\text{0}.\text{33}\frac{A_{k}}{\overset{\text{2}}{\underset{n}{D}}}\right)}^{\text{0}.\text{72}}\right]}$$ (7)

where $\rho_{s}$ is the density of the specimen, $\rho_{f}$ the density of the fluid and g is the acceleration due to gravity. The sum of squared errors (SSE) was calculated to be 0.0089. A good fit of the exponential model was quantified with a coefficient of determination ($R^{\text{2}}$) of 0.75. The root mean square error (RMSE) quantifies the size of the residuals to 0.0028. A lower 90% confidence bound can be derived from the mean value (eq. (7)) in the form $\overset{*}{\underset{-\text{9}\text{0}\%}{\tau }}=\overset{*}{\underset{mean}{\tau }}-\text{1}.\text{6}\text{4}\cdot RMSE$ and can serve as a design threshold for the initiation of motion.

The critical Shields parameter required for the initiation of motion is reduced by an increase of the relative surrogate frontal area. This observation is plausible as an increased approach surface of the surrogate increases the flow-induced load on the specimen. For $\frac{A_{k}}{\overset{\text{2}}{\underset{n}{D}}}<\text{4}$ a comparably strong exponential decrease of the critical Shields parameter is measured. For $\frac{A_{k}}{\overset{\text{2}}{\underset{n}{D}}}>\text{4}$ the further reduction is less pronounced.

## Discussion

The results demonstrate that larger surrogates consistently lead to lower critical shear stresses, indicating a predisposition for stones colonized by kelp to move at lower velocities compared to their uncolonized counterparts. Moreover, the analysis highlights bed roughness as a critical factor, with substantial effects on shear stress thresholds. However, while surrogate size consistently emerged as a significant factor across models, the influence of stone size was contingent on the bed’s roughness. Irrespective of these individual effects, by collapsing our data into the dimensionless Shields parameter, we present a unifying approach to compare shear stress across varying conditions.

### Influence of kelp on the initiation of stone motion

The initiation of motion for individual stones exhibited high variance, which decreased steadily with increasing surrogate size. While the varying shapes of stones contributed to differences in their stability, this effect also diminished as surrogate size increased. This homogenization is a result of the interplay between driving and resisting forces demonstrated by the derived Shields parameter. Surrogate size also affected its dynamic motion and deflection (Fig 6). As a result the vegetation undergoes greater displacement and thus appears more flexible when the ratio between plant size and characteristic wavelength increases, which agrees with previous experiments [32]. The dynamic motion results in fluctuating drag forces, where the total drag force, which consists of the drag on both the stone and the surrogate, becomes increasingly influenced by the surrogate as its size increases.

Under unidirectional flow, such fluctuations in drag force were less pronounced, resulting in even greater homogenization under these conditions (Fig 4). Nonetheless, as the stone volume increases, the resisting forces likely become more substantial, diminishing the relative influence of the surrogate (Fig 4). These findings underscore the critical role of kelp coverage, leading to a homogenization of threshold values for stone movement – an aspect not previously addressed in the literature on kelp-induced sediment transport.

The increased drag forces generated by larger kelp surrogates also led to an initiation of motion under lower energy levels compared to smaller surrogates. A similar effect was observed for large kelp or seaweed fronds in the field, both under waves [33,34] and currents [13,35]. It should be noted that buoyancy, as considered in these studies, becomes a decisive factor for the initiation of motion when the mass ratio of kelp to stone reaches approximately 3:1 [14]. It can be neglected for the surrogates used here, and thus juvenile kelp, as mass ratios did not exceed 1:2. Other forces relevant for the interaction of kelp with hydrodynamics are tension and inertia [36]. They occur when bending of the kelp leads to its full extension and drag and friction continue to act, thus stretching the kelp’s tissue.

### Forces and dynamics within kelp-stone systems

**Fig 8:**
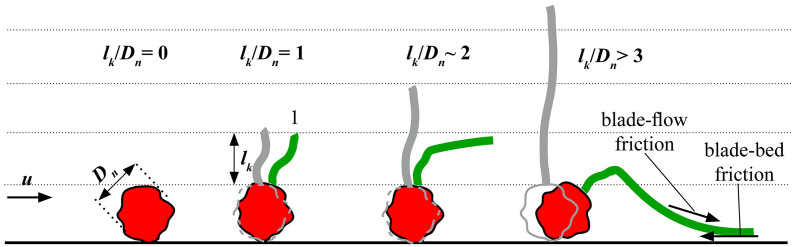
Schematic dynamic response of specimens with respect to the surrogate length.

The correlation between the Shields parameter and relative surrogate size (Fig 7) can be explained by the motion behaviour of the stones with the varying surrogate length. Without surrogate ($\frac{l_{k}}{D_{n}}=\text{0}$) the stone moves by sliding or rolling. With short surrogates ($\frac{l_{k}}{D_{n}}=\text{1}$), they act like a stiff cantilever (Fig 6) and the stone is first tilting in flow direction which leads to a reduction of the frontal area and then finally slides over the bed. With increasing surrogate length ($\frac{l_{k}}{D_{n}}\sim \text{2}$) first the surrogate bends (dependent on the blade stiffness), then the stone tilts in flow direction and then slides over the bed. With further increasing surrogate length, the friction force on the surrogate increases and the critical Shields parameter reduces further. Long surrogate lengths ($\frac{l_{k}}{D_{n}}>\text{3}$) show the same failure mechanism. Additionally, the friction between surrogate and bed surface increases as the surrogate now has a larger surface area in contact with the bed itself. This bed contact of the surrogate was observed for most of the specimen, but could not be quantified in the video analysis (Fig 6) as this analysis was only possible on a kelp-stone system that was fixed in position which prevented stone tilting. Although the frictional force of the flow (blade-flow friction) increases as the length of the surrogate increases, the stabilising frictional force between the surrogate and the bottom (blade-bed friction) increases the stability of the specimen (Fig 8). It is important to note that the described motion behaviour is driven by the bottom flow direction. In oscillatory flows under waves the motion is changing and therefore, the specimens move back under a wave trough and forth under a wave crest.

The described failure mechanisms were observed in both the flow and wave experiments, which were conducted in different laboratory facilities for technical reasons and with different surrogate material to match existing datasets (see Limitations of the study). Irrespective of these differences in experimental setup, the similarity in failure mechanism suggests a wider applicability of this process. In scenarios where the $l_{k}/D_{n}>\text{3}$, similar behaviour has been observed under natural conditions. Kudrass [32] demonstrated by experiment that increased friction can raise the movement threshold, although these effects were more pronounced in mature kelp.

### Influence of the bed condition

Differences in initiation of motion were also observed between different bed conditions with specimens moving at lower critical shear stresses on smooth and sloping beds. Bed roughness affects frictional forces due to the number of contact points between the specimen and the bed. Specimens on flat concrete surfaces rest on a relatively small contact area against a smooth and even surface. In contrast, gravel beds are uneven and substantially rougher. Larger niches formed by the increased bed roughness result in stones having multiple points of contact on these surfaces, which increases friction. Additionally, the stone’s centre of gravity is positioned lower relative to the bed’s surface compared to a smoother bed. The resulting interlocking of stones provides further stabilisation, requiring higher forces, e.g. resulting from larger bottom velocities, to initiate movement [37]. As a result, there was no effect of stone size on critical shear stress for the specimens on the gravel bed. On the concrete bed, however, larger stones ($D_{n}=\text{2}\text{2}\text{mm}$) led to 20% higher critical shear stress (Fig. 4).

Although the larger stones led to a 1.9 times greater frontal area and thus higher drag forces, this effect was counteracted by the increase of resisting forces based on gravity and friction. The hydrodynamic drag force, $F_{d}$, scales linearly with the frontal area $A_{k}$, as expressed by the standard drag relationship


$$F_{d}=\text{0}.\text{5}\rho_{f}u^{\text{2}}C_{d}A_{k}$$ (8)

Where u is a characteristic velocity and $C_{d}$ is the drag coefficient. In contrast, the frictional resistance force, $F_{f}$, is governed by the normal force $F_{n}$, which is primarily determined by the submerged weight of the stone and gravity, such that $F_{f}\sim F_{n}$. Drag therefore acts as a destabilising force promoting motion, whereas friction represents a stabilising force resisting motion. Consequently, an increase in stone diameter $D_{n}$ leads to an increase in frontal area and thus to a higher destabilising drag force. At the same time, the increase in stone size results in a greater weight and normal force, which enhances frictional resistance. The increase in drag with increasing $D_{n}$ is therefore counterbalanced by the concurrent increase in friction, leading to a compensating effect between destabilising and stabilising forces.

It was expected that specimens on inclined concrete surfaces would exhibit greater instability compared to those on flat surfaces. On an inclined surface, the gravitational force is divided into two components, one acting perpendicular to the surface that stabilises the specimen and another acting parallel to the surface that promotes movement. Consequently, inclination reduces resisting forces and would lead to earlier movement. However, our data showed that the overall influence of inclination is relatively minor (Fig. 5). Only an inclination angle of 15° showed a significant impact on the initiation of motion, whereas smaller inclination angles exhibited no difference in motion initiation compared to a smooth bed across any of the surrogate lengths tested. In our setup, the destabilising component of gravity acted perpendicular to the drag force which may have led to negligible effects. It is hypothesised that the effect may be more pronounced if slope and orientation of force coincide.

### Limitations of the study

The experiments were conducted with kelp surrogates to have full control over their dimensions which allowed a systematic analysis of their impact on specimen movement. For simplicity, only one kelp surrogate per stone was used, the width of the surrogates was kept constant and only surrogate length was modified to mimic kelp growth. This, however, does not correspond to natural conditions where both length and width of a kelp blade will change during blade growth and stones may be colonised by multiple juvenile kelp specimens. A surrogate width of 2 cm was chosen, which corresponds to the width of a 10 cm long juvenile kelp blade (F. Stahl, pers. comm.). As a result, the actual frontal area of shorter natural kelp is likely smaller than that of the surrogate used in our experiment. To address this discrepancy, our data analysis incorporated the frontal area present in the model, ensuring that the results are applicable to kelp heights of less than 10 cm. The frontal area approach may also enable consideration of multiple kelp juveniles on a single stonewhich are expected to act in unison on stone movement and their joint frontal area could thus be applied as kelp frontal area $A_{k}$. The success of holdfast attachment to the surrounding ground will then, however, also depend on factors other than stone movement, such as competition among juveniles and their location within the juvenile cluster.

The surrogates were produced from geotextile following the approach by Paul and Henry [21]. To ensure broad applicability of our results across different kelp species, we used distinct surrogate materials for the flow and wave treatment experiments. The objective was to align these surrogates with the traits of various *Laminaria sp.*, using existing datasets for comparison (Table 1). However, it is important to note that the biomechanics data across these datasets were obtained through varying methodologies, resulting in discrepancies in values for flexural rigidity between the two measurement methods [22]. This discrepancy limits direct comparisons of the values between methods. Despite this challenge, it is noteworthy that within the same method, the similarity between the kelp and surrogate materials was well maintained. Furthermore, the results from the two surrogate materials matched closely, suggesting that any potential effects on the study's outcomes are very small and likely negligible compared to other factors, such as surrogate size and bed roughness. Additionally, to incorporate the dataset from MarGate near Heligoland, i.e., a potential Green Gravel location [17], a surrogate material was specifically matched using the same method for obtaining biomechanical data. While this inclusion enriches our study by connecting it to local ecological restoration initiatives, it also underscores the challenge of standardizing methodological approaches across diverse datasets.

Further potential limitations of this study include possible inaccuracies during the experimental setup for the flow tests that may have influenced the results. For instance, due to the limited width of the flow facility, the specimens occasionally experienced additional friction from the flume walls, particularly affecting surrogate sizes of 8–10 cm. This friction was an isolated occurrence that could not be entirely prevented. The very low surface roughness of the acrylic glass walls ($k_{b}\sim \text{0}.\text{0}\text{0}\text{1}\text{5}\text{mm}$) indicates a low influence. Additionally, for smaller surrogate sizes (2–4 cm), instances were observed where the stones tipped over, causing the surrogate to come into contact with the flume bed, thereby introducing additional friction similar to blade bending for the case $\frac{l_{k}}{D_{n}}>\text{3}$ (Fig 8). Again, these occurrences were limited and not precisely quantified.

While the specimens for the flow experiments were placed with a predefined orientation, the orientation of specimens for the wave experiments varied with each new setup as it was not deliberately controlled. This variability could impact movement behaviour, as it alters the exposed surface area of the specimens (stone and surrogate) and their positioning on the gravel bed, potentially affecting contact areas and interlockings. This approach is deemed acceptable since, under real-world conditions, particularly when using the Green Gravel method, the orientation of stones is not specifically controlled. Moreover, natural wave directions and flow conditions also vary, which would likely produce similar effects.

The flow experiments were conducted with individual specimens in full replication while for the wave experiments all 150 specimens were exposed to the wave conditions simultaneously. This approach aligns with sample replication in field ecology and while it is acknowledged that this approach bears limitations concerning potential unknown influences [28], this was deemed acceptable as chemical or biological conditions were considered irrelevant since we worked with inert material. The physical conditions which were at the core of this experiment, in contrast, were fully controlled following established protocols in standard engineering practise.

### Practical implications

The practical implications of our study highlight several key considerations for the successful implementation of the Green Gravel method in restoring kelp forests. One of the primary factors influencing success is ensuring that the colonized stones remain in place long enough for the kelp to securely anchor itself to the surrounding seafloor [9]. This underscores the importance of carefully assessing both substrate characteristics and hydrodynamic conditions at potential restoration sites.

Site selection is simplified by our observation of a minimal impact of bed slope on stone stability, with our study covering slopes up to 15°. Although steeper slopes do exist, our findings suggest that a wide array of areas could potentially be suitable for kelp restoration under this method, thus broadening the scope for restoration projects.

While we defined the initiation of motion as the spatial displacement of stones, it is important to note that we also observed movement in the form of tilting or rocking preceding displacement. This type of movement may hinder the expansion of holdfasts and eventually lead to displacement as the kelp grows and the force balance changes. Therefore, the threshold values derived from our study should be used conservatively when evaluating site suitability concerning hydrodynamic conditions.

Our findings indicate that the texture of the substrate plays a significant role in the stability of the specimens. Specifically, a rough surface can enhance specimen stability and reduces the impact of stone size on the initiation of motion. This partially contradicts Earp et al. [10], who suggested that smaller stones are displaced at lower critical shear stresses compared to larger ones. Our data propose that the ratio of kelp size to stone diameter is a more critical determinant of the Shields parameter and, thus, the initiation of motion. As the kelp grows, this ratio changes, suggesting that focusing on absolute stone size may not be crucial throughout a restoration project. Furthermore, smaller stones may present several advantages, particularly on rough substrates. Their ability to lodge into cracks and conform to the overall substrate roughness can lead to reduced movement. Additionally, the smaller stones facilitate quicker envelopment by attachment organs, enabling more effective anchoring to the substrate.

While our study provides valuable insights into the physical dynamics of stone stability with attached kelp, it is important to recognize that this research addresses only one specific aspect of the Green Gravel method as a complex restoration approach. The success of the Green Gravel method depends on a multitude of interacting factors beyond the scope of our laboratory experiments. In our controlled environment, we focused on the mechanical interactions between stones and inert kelp surrogates, which allowed for precise control over physical variables. The natural environment encompasses diverse conditions that can affect juvenile density, holdfast development, and ecological interactions, all of which pose challenges to our study's applicability in predicting stone motion or successful attachment *in situ*. We acknowledge the importance of these biological and ecological factors, and we encourage practitioners to consider them alongside our findings. While the variables tested here are crucial for understanding some mechanical aspects, the ecological success of the Green Gravel method will also depend on factors influencing holdfast robustness and attachment in variable natural settings.

In conclusion, our study provides insights into the dynamics of kelp-stone systems within the framework of the Green Gravel method for kelp forest restoration. The findings underscore the importance of considering multiple factors, such as bed roughness and kelp size, in understanding the threshold for stone movement, but also highlight the low relevance of bed slope. By revealing the key of the Shields parameter as a unifying metric, we offer a comprehensive approach to evaluating the impact of shear stress across diverse conditions. The notion that smaller stones, particularly on rough surfaces, may contribute to greater stability and effective kelp anchoring broadens the potential applicability of Green Gravel projects across various sites. Moreover, our work highlights the intricate interplay of forces inherent in kelp-stone systems and the significance of kelp size in influencing these dynamics, moving beyond the simplistic assessments of stone size alone. Ultimately, these insights are instrumental for refining site selection and project design alongside the consideration of other environmental conditions [38], ensuring that restoration efforts are both effective and sustainable. By enhancing our understanding of the mechanistic processes underpinning kelp restoration, this study contributes to the broader objectives of marine habitat conservation and ecosystem recovery.
